# Development of FRET-based cap-snatching endonuclease assay

**DOI:** 10.1128/spectrum.03289-24

**Published:** 2025-03-31

**Authors:** Jeeva Subbiah, Austin Royster, Sheema Mir, Mohammad Mir

**Affiliations:** 1 College of Veterinary Medicine, Western University of Health Sciences 6645, Pomona, California, USA; Kumamoto Daigaku, Kumamoto, Japan

**Keywords:** bunyavirus, endonuclease, virus replication

## Abstract

The order *Bunyavirales* consists of over 300 species of segmented, negative-strand RNA viruses. These viruses have a tri-segmented genome (S, M, and L segments) that encodes the nucleocapsid protein (N protein), glycoprotein precursor, and RNA-dependent RNA polymerase (RdRp), respectively. The RdRp is a large protein (~420 kDa) responsible for synthesizing viral mRNA and replicating the viral genome. Bunyaviruses initiate transcription of viral mRNA through a unique cap-snatching mechanism. During this process, the N-terminal endonuclease domain of the RdRp cleaves host cell mRNA at the 5′ terminus and uses the resulting capped mRNA fragment as a primer. This endonuclease domain exhibits a highly conserved structural architecture and is essential for establishing viral infection in host cells. Therefore, the N-terminal endonuclease domain represents a promising target for therapeutic intervention against Bunyaviruses, particularly at the early stages of the virus replication cycle. In this study, we developed a highly sensitive fluorescence resonance energy transfer-based *in vitro* assay to quantitatively examine the activity of the bacterially expressed and purified endonuclease domain of hantavirus RdRp. A 20-nucleotide synthetic RNA, labeled with a 6-FAM fluorophore at the 5′ end and an Iowa Black quencher at the 3′ end, generated a significant dequenched fluorescence signal upon cleavage by the purified endonuclease domain. Kinetic analysis revealed a half-life (*t*_1/2_) of approximately 3 min for the reaction, achieving a signal-to-background ratio of approximately 31.

**IMPORTANCE:**

Viruses belonging to the order *Bunyavirales*, including Hantaviruses, Crimean–Congo hemorrhagic fever virus, Rift Valley fever virus, Severe Fever with Thrombocytopenia Syndrome Virus, and La Crosse encephalitis virus, cause severe human illnesses with mortality rates in certain outbreaks reaching 50%, 10%–40%, 10%–20%, 6%–30%, and 1%, respectively. Currently, there are no Food and Drug Administration-approved vaccines or antiviral therapeutics available for these viruses. The highly efficient and cost-effective fluorescence resonance energy transfer-based *in vitro* endonuclease assay, having a quantitative fluorescence readout, can be optimized for high-throughput screening of chemical libraries to identify chemical inhibitors for the Bunyavirus cap-snatching endonuclease. The assay will be of critical importance for antiviral drug discovery against numerous negative-strand RNA viruses using cap-snatching mechanism for transcription initiation and replication of the RNA genome.

## INTRODUCTION

**Fig 1:**
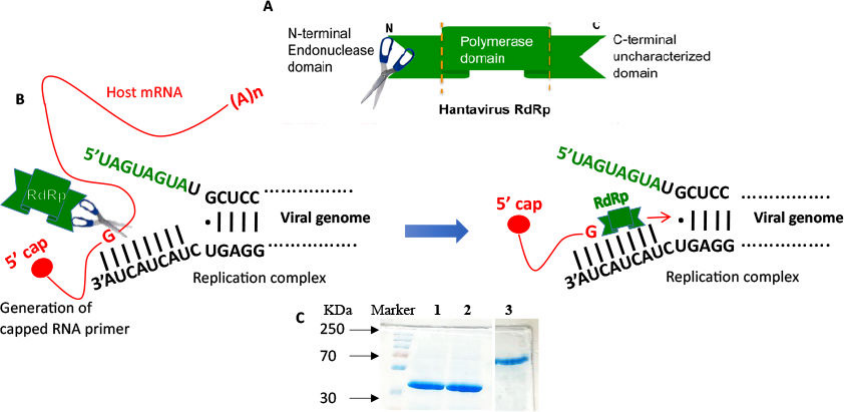
(A). Structural domains of the hantavirus RdRp. (B) The N-terminal cap-snatching endonuclease domain (scissor) cleaves the host mRNA (red line) from the 5′ terminus and uses the cleaved capped mRNA fragment as primer. (C) Bacterially expressed and purified endonuclease domain of Sin Nombre virus (SNV) RdRp (lane 1), endonuclease D97A mutant (lane 2), and N protein (lane 3).

The Bunyaviruses, Arenaviruses, and Orthomyxoviruses contain negative-sense RNA genomes, and their infections lead to significant human diseases. For example, the highly pathogenic and clinically important viruses, such as Hantaviruses, Rift Valley fever virus, Crimean–Congo hemorrhagic fever virus (CCHFV), Lassa fever virus, and influenza virus, cause serious human illness, with no cure at present for most of these viral infections. More than 300 Bunyavirus species contain tri-segmented RNA genome (S, L, and M segment) that encode nucleocapsid protein (N protein), RNA-dependent RNA polymerase (RdRp) and glycoproteins Gn and Gc, respectively. The Bunyavirus RdRp is a large protein of 240 to 420 kDa, carrying out viral mRNA synthesis and replication of the viral genome in the cellular cytoplasm. In comparison, the RdRp of the influenza virus from the Orthomyxoviridae family is composed of three subunits and carries out transcription and replication of the viral genome in the cellular nucleus. The replication being either cytoplasmic or nuclear in nature, these viruses initiate the viral mRNA synthesis by a unique “cap-snatching” mechanism, during which host cell mRNAs are cleaved close to the 5′ terminus by the endonuclease activity of the RdRp, and the resulting capped mRNA fragments are used as primers to initiate the viral mRNA synthesis (Fig. 1). The Bunyavirus RdRp contains the N-terminal cap-snatching endonuclease domain, a central catalytic domain, and a large C-terminal domain of unknown function (1, 2) (Fig. 1A). The highly conserved endonuclease domain of the *Bunyavirus* RdRp shares a type II endonuclease α/β architecture similar to the N-terminal endonuclease domain of influenza virus PA subunit (2–7). The X-ray crystal structures of the cap-snatching endonuclease domain from La Crosse *Orthobunyavirus* (LCV), Andes hantavirus, and influenza virus reveal a similar overall fold and identical two metal-binding active sites (3, 8–10). We previously reported that the hantavirus RdRp preferentially cleaves a host cell mRNA at a "G" residue, located 14 nucleotides downstream of the 5′ cap, and uses the resulting 14-nucleotide capped RNA fragment, which contains a 3′ "G" residue, as a primer to initiate transcription (11). We showed that hantaviruses preferentially snatch caps from non-sense mRNAs that are targeted to P-bodies for degradation (11). Other investigators have also shown that LCV, hantavirus, and influenza virus use capped RNA primers of 10–14 nucleotides in length, containing a 3′ “U,” “G,” and “C or A” residues, respectively (11–13). In addition, we also reported that the hantavirus N protein binds to the C-terminal uncharacterized domain of the hantavirus RdRp. This interaction between the N protein and RdRp is essential for the synthesis of hantavirus mRNA and the replication of the viral genome (14). Other investigators later demonstrated that N protein–RdRp interaction is also required for the viral mRNA synthesis of CCHFV, another member of the *Bunyavirales* order (15). The endonuclease activity of RdRp plays a crucial role in Bunyavirus replication and the establishment of infection in the host cell. Disabling this activity would significantly impact virus replication and its spread to neighboring cells. Therefore, the cap-snatching endonuclease domain of RdRp represents a promising target for therapeutic intervention against deadly viruses from the *Bunyavirales* order. Given the highly conserved overall structural architecture of Bunyaviruses, chemical inhibitors targeting the endonuclease domain of RdRp are likely to exhibit broad-spectrum antiviral activity.

In this article, we report the development of highly efficient fluorescence resonance energy transfer (FRET)-based endonuclease assay to monitor the endonuclease activity of bacterially expressed and purified endonuclease domain of hantavirus RdRp. This assay can be used in high-throughput mode to screen the chemical libraries for the identification of chemical inhibitors for the hantavirus cap-snatching endonuclease. The broad-spectrum applicability of the assay for other Bunyaviruses will require testing of their endonuclease domains for activity using the FRET assay developed in this manuscript.

## RESULTS

### Purification of Sin Nombre virus (SNV) N protein, RdRp endonuclease domain, and its point mutant

SNV N protein, RdRp endonuclease domain, and its D97A point mutant were expressed in *E. coli* as C-terminally His-tagged fusion proteins (see Materials and Methods for details). The proteins were purified by NiNTA chromatography on AKTA pure protein purification system (GE Healthcare), using a native purification procedure. As shown in Fig. 1C, the purified N protein, endonuclease domain, and its point mutant were free of detectable heterologous bacterial proteins. The electrophoretic mobilities of N protein and endonuclease domain were consistent with their expected molecular masses of ~54 and ~32 kDa, respectively.

### The purified endonuclease domain of SNV RdRp shows RNA endonuclease activity *in vitro*

The cap-snatching endonuclease domains, expressed and purified from various viruses within the *Bunyavirales* order, including Andes hantavirus (8), La Crosse *Orthobunyavirus* (2), lymphocytic choriomeningitis virus (3), and Lassa virus (16), have demonstrated nonspecific RNA endonuclease activity *in vitro*. We next wanted to determine whether the purified endonuclease domain of the Sin Nombre hantavirus RdRp exhibits biological activity.

**Fig 2:**
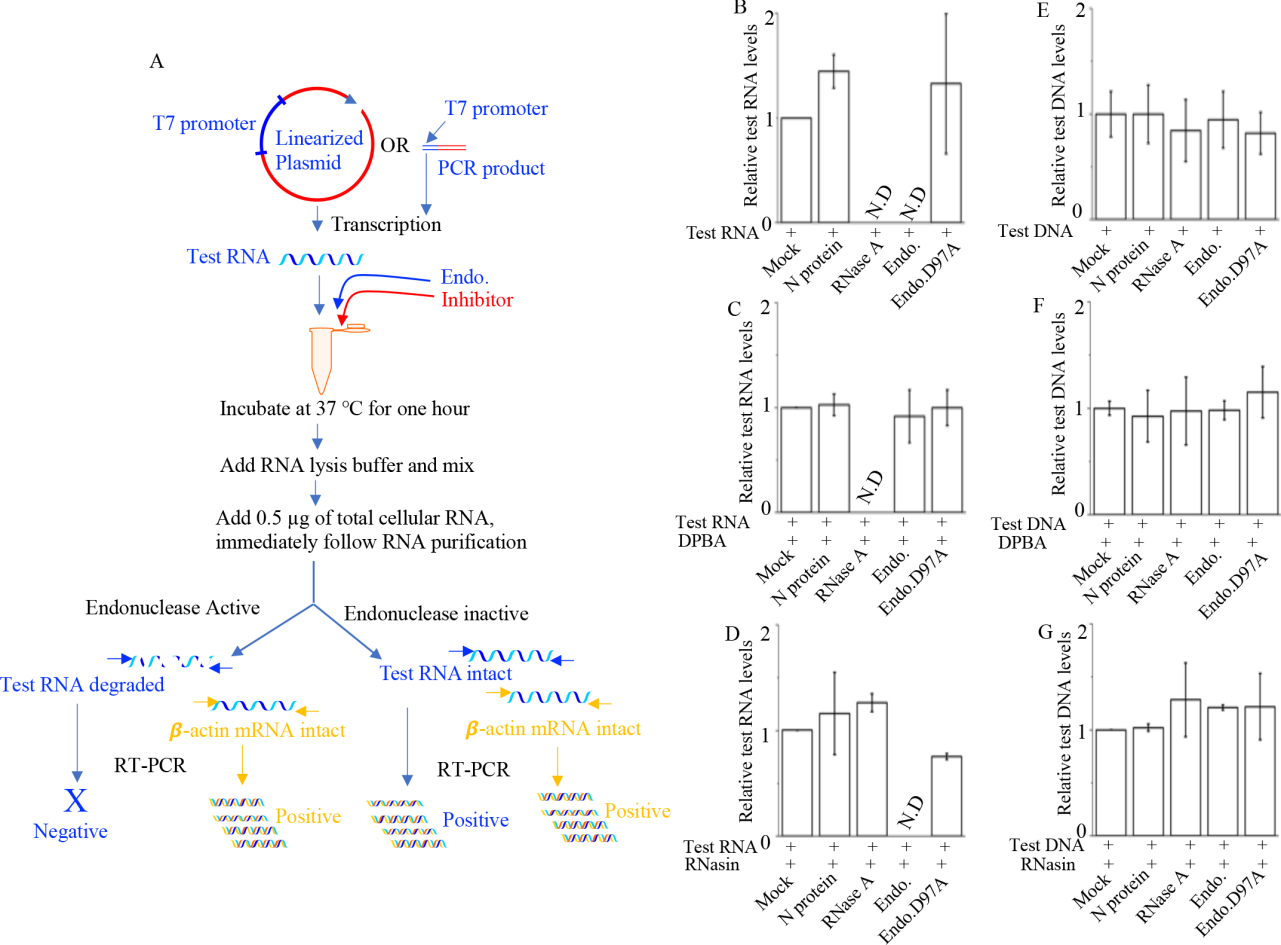
Examination of RNA endonuclease activity by RT-qPCR: (A) A schematic showing a linearized plasmid containing a T7 promoter or a PCR product with flanking T7 promoter is transcribed *in vitro* using a T7 transcription kit. The resulting RNA (test RNA) is purified and incubated with the endonuclease domain (endo.) in the absence or presence of the inhibitor at 37°C for 1 h. RNA lysis buffer is added to terminate the reaction, followed by the addition of 0.5 µg of total cellular RNA before the RNA purification is carried out. The test RNA is quantified by RT-qPCR using β-actin as internal control. (B–D). Test RNA (350 ng) was incubated with either phosphate-buffered saline (PBS; mock) or bacterially expressed and purified N protein (50 nM), RNase A (50 U), endonuclease domain, shown as Endo. (50 nM), the endonuclease D97A point mutant, shown as Endo.D97A (50 nM), in RNA digestion buffer at 37°C for 1 h in the absence (B) or presence of 200 µM DPBA (C) or 100 U of RNasin (D). The purified proteins used in this assay were dissolved in 1× PBS. The purified test RNA was quantified by RT-qPCR using β-actin as internal control. The quantified test RNA levels were normalized relative to mock and plotted along the *Y*-axis. (E–G). The experiment was repeated exactly as in (B)–(D), except that the PCR-amplified DNA was used instead of RNA. After the incubation of reaction mixtures at 37°C for 1 h, the DNA was purified by a plasmid min-prep kit without the addition of RNA lysis buffer or 0.5 µg of total cellular RNA, as shown in(A). The eluted DNA (5 µL) from each sample was quantified by real-time PCR, and the DNA levels were normalized to mock control (G) and plotted along the *Y*-axis.

A 300-nucleotide-long test RNA was synthesized *in vitro* using a T7 transcription kit, as previously described (17–19). Briefly, a PCR product with a flanking T7 promoter was generated from the pCDNA3.1(+) plasmid using two opposing primers. The PCR product was gel purified and used as a template for the T7 transcription reaction, as mentioned in the Materials and Methods section. Alternatively, a plasmid containing a T7 promoter could be linearized using a restriction enzyme downstream of the T7 promoter, allowing the linearized plasmid to serve directly as a template in the *in vitro* T7 transcription reaction (Fig. 2A). The synthetic RNA was purified using the RNeasy kit (Promega) following the manufacturer’s instructions. Purified RNA (350 ng) was then incubated with either a bacterially expressed and purified endonuclease domain, the endonuclease D97A point mutant, or the N protein at a concentration of 50 nM, or with 50 U of RNase A, in 50-µL of RNA digestion buffer (10 mM Tris-HCl, pH 8.0, and 1 mM MnCl₂) at 37°C for 1 h. Reactions were terminated by adding 300 µL of RNA lysis buffer from the RNA purification kit (Zymo), followed by incubation at room temperature for 10 min. Additionally, 0.5 µg of total RNA extracted from HEK293T cells was added to each reaction tube, immediately followed by RNA purification using the RNeasy kit (Fig. 2A, schematic). Test RNA in each tube was quantified by real-time PCR (relative quantification method) using a primer set complementary to the 5′ and 3′ ends of the test RNA, with β-actin serving as an internal control. Selective degradation of the test RNA indicated active endonuclease activity of the input protein in the reaction (see Fig. 2A schematic). The reactions were performed either in the absence (Fig. 2B) or presence of DPBA (Fig. 2C), a known inhibitor of hantavirus cap-snatching endonuclease (8), or RNasin (Fig. 2D), an inhibitor of RNase A. As shown in Fig. 2B, both the endonuclease domain and RNase A completely degraded the test RNA, rendering it undetectable in this assay. However, neither the N protein nor the D97A endonuclease mutant exhibited RNase activity. Treatment with DPBA (Fig. 2C) selectively inhibited the endonuclease domain, while RNasin (Fig. 2D) specifically inhibited RNase A.

The experiment was repeated exactly with double-strand DNA (Fig. 2E and F). Briefly, 300 ng of gel-purified PCR product (~330-nucleotide long) was incubated with the endonuclease domain, the endonuclease D97A point mutant, or the N protein at a concentration of 50 nM, or with 50 U of RNase A, in the absence (Fig. 2E) or presence of either DPBA (Fig. 2F) or RNasin (Fig. 2G) at 37°C for 1 h, as mentioned above. The DNA from each tube was then purified using a plasmid mini-prep kit (Thermo Scientific). The purified DNA levels in each tube were quantified by real-time PCR and normalized to the mock control. As shown in Fig. 2E through G, none of the proteins, whether in the presence or absence of DPBA or RNasin, caused any degradation of the tested double-stranded DNA. This clearly demonstrated that the endonuclease domain of the Sin Nombre virus RdRp has RNA-specific endonuclease activity.

### Cleavage of FRET RNA by the hantavirus endonuclease domain

**Fig 3:**
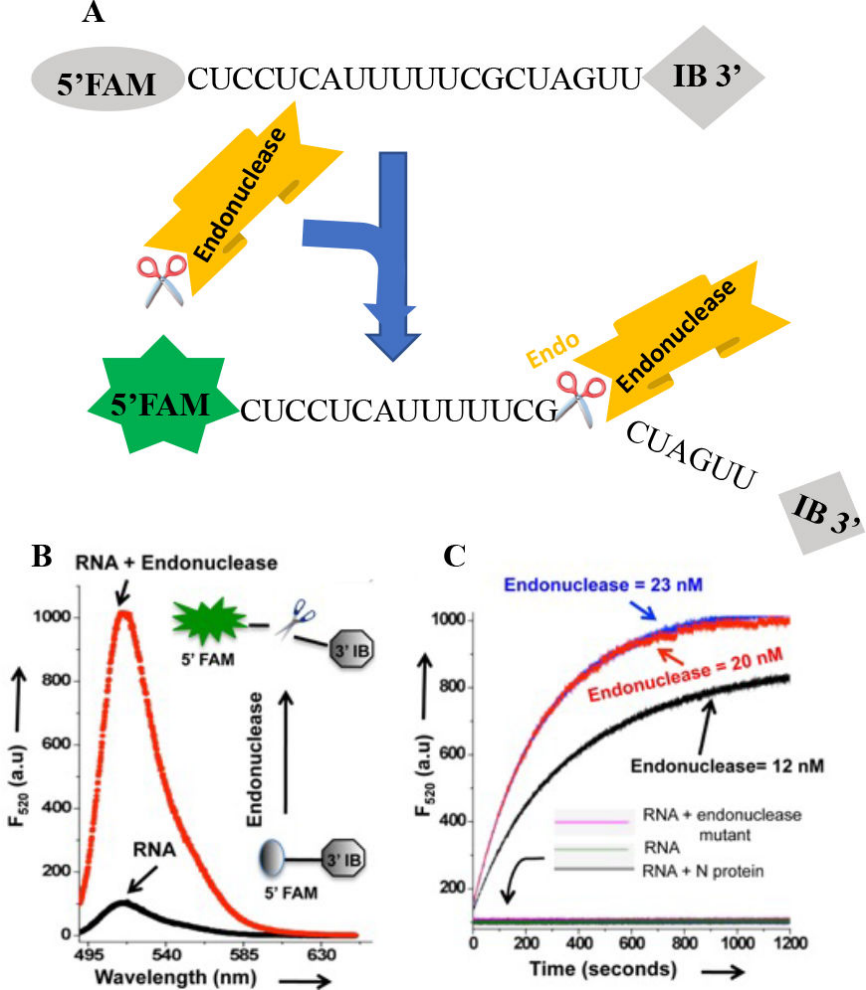
FRET-based endonuclease assay. (A) A 20-nucleotide-long RNA (5′FAM-CUCCUCAUUUUUCGCUAGUU-IB3′), labeled with 6-FAM fluorophore at the 5′ terminus and Iowa Black (IB) quencher at the 3′ terminus, is referred to from here onward as FRET RNA. The FRET RNA is cleaved by the endonuclease domain, generating the dequenched fluorescence signal. (B) FRET RNA sample (120 nM) in digestion buffer (10 mM tris HCl, pH 8.0, and 1 mM MnCl2) was excited at 465 nm, and the emission spectrum was recorded from 495 to 650 nm (black line). The FRET RNA was then incubated with the purified cap-snatching endonuclease domain (20 nM), and the spectrum in the same wavelength range was again recorded (red line). The cartoon on the right shows that the treatment of FRET RNA with endonuclease domain (shown as sizer) causes the RNA cleavage, resulting in the generation of dequenched fluorescence signal (green). (C) The FRET RNA sample (120 nM) in digestion buffer was treated with either N protein (20 nM) or mutant endonuclease domain having D97A point mutation (20 nM) or with increasing concentrations (12, 20, or 23 nM) of the wild-type endonuclease domain, and the fluorescence intensity at 520 nm was recorded over time.

A 20-nucleotide-long RNA was synthesized, and its 5′-terminus was labeled with a fluorophore 6-FAM (6-carboxyfluorescein). The 3′ terminus was labeled with a quencher Iowa Black (IB) (Fig. 3A). The synthetic FRET RNA (5′FAM-CUCCUCAUUUUUCGCUAGUU-IB3′) contained a “G” residue 14 nucleotides from the 5′ end. This “G” residue was incorporated due to preferential cleavage of the host mRNA by the hantavirus RdRp at a “G” residue 14 nucleotides down of the 5′ terminus (20). We previously reported that hantaviruses efficiently snatched this sequence from a transcript expressed in the infected cells from a transfected plasmid (20). This sequence was cleaved at the 14th “G” residue from the 5′ terminus and used as primer for transcription initiation (20). Since bacterially expressed and purified endonuclease domains of multiple Bunyaviruses and influenza A virus nonspecifically cleaves the RNA *in vitro* (2–4, 8, 13, 16, 21), it is possible to use any RNA sequence in the FRET RNA endonuclease assay. We preferred to use the above sequence based on our previous studies (14, 20, 22). Due to its proximity, the 3′ quencher (Iowa Black) quenches the fluorescence signal of 6-FAM by the FRET. The FRET RNA was dissolved in Molecular Biology-grade water (Fisher Scientific). A sample of FRET RNA (120 nM) in RNA digestion buffer (10 mM Tris HCl, pH 8.0, and 1 mM MnCl2) was excited at 465 nm, and the fluorescence emission spectrum was recorded from 495 to 650 nm (black line, Fig. 3B). The fluorescence emission peak was observed at 520 nm (Fig. 3B). It is well known that absorption and emission spectra of the free 6-FAM molecule slightly overlap, and the absorption and emission peaks are at 495 and 520 nm, respectively. To avoid the potential fluorescence bleed through, the FRET RNA was excited at 460 nm, 25 nm away from the absorption peak of 495 nm.

The FRET RNA was then incubated with the purified cap-snatching endonuclease domain at a fixed concentration of 20 nM in the digestion buffer at room temperature (25°C) for 20 min, and the fluorescence emission spectrum in the same wavelength range was again recorded (red line, Fig. 3B). It is evident that the fluorescence quantum yield of 6-FAM was significantly increased after incubation with the purified endonuclease domain (compare black and red spectra in Fig. 3B). We did not observe any noticeable shift in the fluorescence emission peak. The increase in the fluorescence quantum yield is due to the detachment of the fluorophore 6-FAM from the quencher Iowa Black by the endonucleolytic cleavage of the FRET RNA, caused by the endonuclease domain.

### Kinetics of the endonuclease activity

The kinetics of the endonuclease reaction was monitored by incubating FRET RNA (120 nM) with purified endonuclease domain at varying concentrations (12 to 23 nM) while measuring fluorescence intensity at 520 nm (*F*_520_) over time. A plot of *F*_520_ against time displayed a hyperbolic kinetic curve, with a half-life (*t*_1/2_) of approximately 3 min (Fig. 3C). The *t*_1/2_ represents the time required for half of the reaction to be completed. Subsequently, the endonuclease activity of a bacterially expressed and purified endonuclease mutant containing an active site point mutation (D97A) was evaluated using the FRET assay. As shown in Fig. 3C, incubating the mutant (23 nM) with FRET RNA (120 nM) did not produce any fluorescence signal over time, confirming the absence of endonuclease activity. Similarly, the experiment was conducted with bacterially expressed and purified N protein, which also showed no measurable increase in fluorescence over time, further confirming the lack of endonuclease activity.

### RNA-specific endonuclease activity of SNV RdRp endonuclease domain

**Fig 4:**
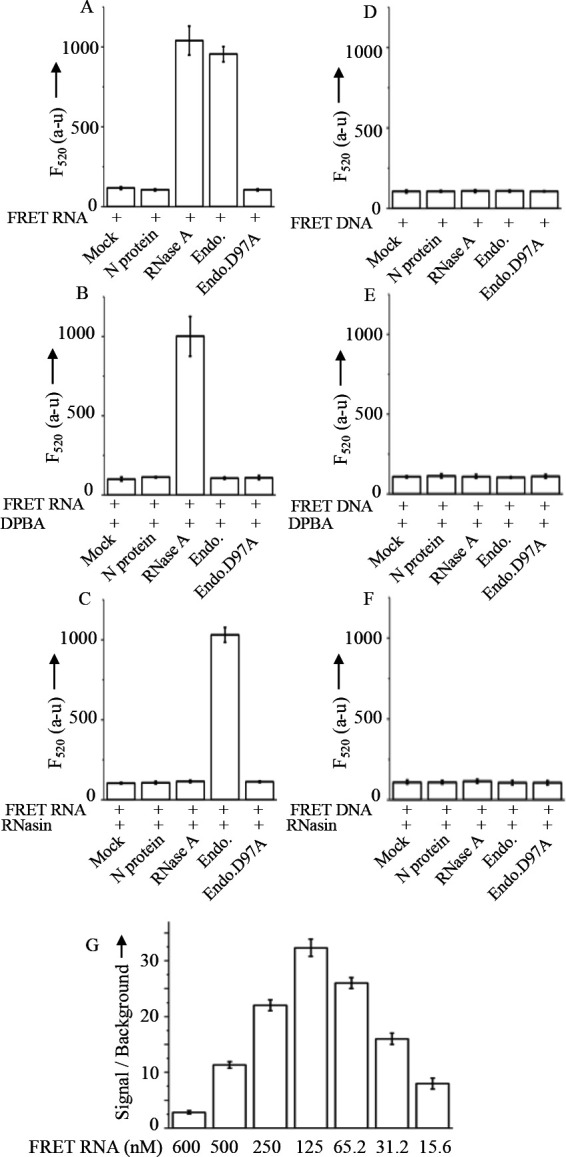
Characterization of endonuclease activity. (A–F) The FRET RNA (A–C) or FRET DNA (D-F) at a concentration of 120 nM in digestion buffer were incubated with either 1× PBS (mock) or 20 nM N protein (negative control) or 50 U of RNase A (positive control) or 20 nM endonuclease domain, shown as an Endo. or 20 nM endonuclease D97A mutant, shown as Endo.D97A, at 25°C in the absence (A, D) or presence of either 200 µM DPBA (B. E) or 100 U of RNasin (C, F). The recorded fluorescence signal (*F*_520_) was plotted along the *Y*-axis. (G) The FRET RNA was diluted from 600 to 15.6 nM and incubated with 20 nM endonuclease domain at 25°C for 30 min. The fluorescence signal of FRET RNA samples treated with endonuclease domain was divided by the corresponding fluorescence signal of FRET RNA samples without endonuclease treatment. The ratio was plotted versus FRET RNA concentration.

We next sought to determine whether the SNV RdRp endonuclease domain specifically cleaves RNA by comparing its endonuclease activity against a single-stranded DNA (ssDNA) molecule of the same length. Briefly, similar to the FRET RNA, a 20-nucleotide-long ssDNA molecule (5′-FAM-CTCCTCATTTTTCGCTAGTT-IB3′) was synthesized by IDT and labeled with a 6-FAM fluorophore at the 5′ terminus and an Iowa Black (IB) quencher at the 3′ terminus. Like the FRET RNA, the ssDNA molecule contained a “G” residue located 14 nucleotides downstream of the 5′ terminus. The FRET DNA molecule was dissolved in Molecular Biology-grade water (Fisher Scientific). Endonuclease reactions, containing 120 nM FRET RNA (Fig. 4, panels A–C) or FRET DNA (Fig. 4, panels D–F) in the digestion buffer were incubated with either 20 nM of N protein, the endonuclease domain, the endonuclease D97A mutant, or 50 U of RNase A at room temperature (25°C) for 20 min in the absence (Fig. 4 panels A, D) or presence of DPBA (Fig. 4 panels B, E) or RNasin (Fig. 4 panels C. F). Fluorescence emission (*F*_520_) was recorded as described in the Materials and Methods section. As shown in Fig. 4A, both the endonuclease domain and RNase A (positive control) produced a positive fluorescence signal, indicating endonucleolytic cleavage of the FRET RNA. This cleavage physically separated the FAM fluorophore from the static quencher, Iowa Black, resulting in the dequenching of FAM fluorescence signal. In contrast, the fluorescence signal of the FRET DNA probe remained unchanged after treatment with N protein, the endonuclease domain, the endonuclease D97A mutant, or RNase A. Consistent with findings in Fig. 2, these results further confirm the RNA-specific endonuclease activity of the SNV RdRp endonuclease domain. Again, consistent with Fig. 2, the treatment with DPBA (Fig. 4B) and RNasin (Fig. 4C) selectively inhibited the cleavage of FRET RNA by the SNV endonuclease domain and RNase A, respectively. These treatments had no impact upon the fluorescence signal of FRET DNA probe (Fig. 4E and F)

### Signal-to-background ratio

With the use of positive and negative controls, Fig. 4A clearly demonstrates that the endonuclease domain of SNV RdRp specifically cleaved the FRET RNA and generated a significant fluorescence readout in comparison to the negative control. The assay can be used for high-throughput screening of chemical libraries to identify the chemical compounds that specifically inhibit the endonuclease activity of SNV RdRp. One of the important criteria for an assay to be used for high-throughput screening of chemical libraries is to have a high signal-to-background ratio, mandating the need to determine the signal-to-background ratio for this FRET-based endonuclease assay. We sought to determine the concentrations of the endonuclease domain and FRET RNA in the endonuclease reaction at which the highest signal-to-background ratio is observed. The FRET RNA was serially diluted from 600 to 15.6 nM and incubated with a fixed concentration of endonuclease domain (20 nM) in the digestion buffer at room temperature for 20 min. The fluorescence signal (*F*_520_) of the reaction mixture was quantified by the spectrofluorometer. The resulting fluorescence signal was divided by the fluorescence signal from untreated FRET RNA control, and the ratio was plotted. It is evident from Fig. 4G that under these conditions, the highest signal-to-background ratio of 31 was observed at 125 nM FRET RNA concentration.

## DISCUSSION

Viruses belonging to the Bunyavirales order, including Hantaviruses, Crimean–Congo hemorrhagic fever virus (CCHFV), Rift Valley fever virus (RVFV), Severe Fever with Thrombocytopenia Syndrome Virus (SFTSV), and La Crosse encephalitis virus (LCV), cause severe human illnesses with mortality rates in certain outbreaks reaching 50%, 10%–40%, 10%–20%, 6%–30%, and 1%, respectively. Currently, there are no FDA-approved vaccines or antiviral therapeutics available for these viruses. This highlights the urgent need for the development of vaccines or antiviral drugs that can be readily accessible to the population during outbreaks. The recent rapid development of mRNA vaccines against SARS-CoV-2 has revolutionized vaccinology, offering hope for the creation of mRNA-based vaccines against medically significant Bunyaviruses. However, their widespread use is limited, primarily due to the need for cold storage and administration by licensed medical professionals. In contrast, the availability and easy access to oral antiviral drugs could play a crucial role in reducing mortality caused by these viral diseases. Therefore, identifying novel targets for antiviral drug discovery remains a top priority in combating these life-threatening Bunyavirus-induced illnesses.

All Bunyaviruses share a common mechanism for transcription initiation and genome replication, during which the L-segment-encoded RdRp cleaves the host cell mRNA using its N-terminal endonuclease domain. The resulting capped mRNA fragment is then used as a primer for viral transcription. Therefore, the N-terminal endonuclease domain represents a promising target for antiviral drug development to inhibit Bunyavirus replication at an early stage in the host cell.

The X-ray crystal structures of multiple negative-strand RNA viruses, including hantaviruses, LCV, LCMV, and influenza A virus (IAV), have revealed a conserved structural architecture for cap-snatching endonucleases. These structures consist of two lobes, with the conserved active site buried within a cavity between them (2–4, 8, 13, 16, 21). Previous X-ray crystallographic studies, along with thermal stability and enzymatic activity data of the Andes hantavirus endonuclease domain, have shown that the residues His36, Asp97, and Glu110 coordinate a manganese ion and are essential for catalysis (8). These residues act cooperatively, as the removal of any one residue disrupts metal ion binding and renders the enzyme inactive (8). By building on this structural data, we aimed to develop a sensitive and cost-effective *in vitro* assay with a quantitative readout to monitor the endonuclease activity of the hantavirus cap-snatching endonuclease domain. Such an assay could be adapted for high-throughput screening of chemical libraries to identify chemical inhibitors targeting the Bunyavirus cap-snatching endonuclease.

The RT-qPCR-based assay (Fig. 2A) involves incubating synthetic RNA with the endonuclease domain, followed by RNA purification and quantification of undegraded RNA using real-time PCR. This multi-step process requires large amounts of synthetic test RNA and yields low levels of undegraded RNA due to inefficient purification methods. Additionally, the assay is costly, labor intensive, and complex, making it unsuitable for high-throughput screening of chemical libraries. Similarly, polyacrylamide and agarose gel-based assays, which use radiolabeled or fluorescently labeled RNA substrates to track cleavage products, are inefficient and lack precise quantification. Their adaptation for high-throughput screening is highly impractical. Surface plasmon resonance (SPR)-based assays, which rely on the binding of undegraded RNA to substrates like complementary DNA, are also time consuming and expensive, further limiting their applicability for high-throughput screening.

However, a 20-nucleotide synthetic FRET RNA, labeled with a 6-FAM fluorophore at the 5′ end and an Iowa Black (IB) quencher at the 3′ end, produced a significant dequenched fluorescence signal upon incubation with the bacterially expressed and purified endonuclease domain of the SNV RdRp (Fig. 3B). Kinetic analysis revealed that the endonuclease domain cleaved the FRET RNA with a *t*_1/2_ of approximately 3 min. While the *t*_1/2_ of enzymatic reactions depends on factors, such as temperature, buffer composition, and pH, most RNA endonucleases exhibit a *t*_1/2_ of 2–5 min under optimal conditions. As shown in Fig. 2C, negative controls, such as the N protein and the endonuclease D97A mutant, failed to cleave the FRET RNA, as evidenced by the absence of a dequenched fluorescence signal over time. This observation is consistent with previous reports that demonstrated a lack of endonuclease activity in the Andes hantavirus D97A mutant (8).

To enable high-throughput screening assays, a positive control is essential. Therefore, the FRET endonuclease assay was repeated using RNase A as a positive control. As shown in Fig. 4A, the endonuclease activity of the hantavirus endonuclease domain was comparable to that of RNase A. Additionally, Fig. 2 and 4 highlight the RNA-specific activity of the SNV RdRp endonuclease domain. The assay was validated using two well-known inhibitors, DPBA and RNasin, for hantavirus endonuclease and RNase A, respectively (Fig. 4A through C). DPBA chelates the manganese ion, which is required for the catalytic activity of hantavirus endonuclease. Thus, the treatment with DPBA dramatically inhibits the endonuclease activity of SNV RdRp endonuclease (Fig. 4). This was further verified by the D97A endonuclease mutant that fails to bind the manganese ion at the active site and, hence, lacks the endonuclease activity.

Under the reported experimental conditions, 120 nM FRET RNA and 20 nM purified endonuclease domain in digestion buffer, incubated at room temperature for 20 min, the FRET endonuclease assay achieved a signal-to-background ratio of 31 (Fig. 3B). Due to its simplicity, cost effectiveness, quantitative output, ease of execution, and efficiency, this assay is highly suitable for high-throughput screening of chemical libraries. Although the reaction conditions and appropriate controls are well established, further optimization of this assay is required to ensure it meets statistical criteria for high-throughput screening of chemical libraries to identify inhibitors targeting the Bunyavirus RdRp endonuclease. The suitability of an assay to be used for high-throughput screening is typically assayed by determining two statistical parameters, the “coefficient of variation,” which compares the standard deviation of the measured samples to the mean, and the “z” value of the assay, which is also indicative of reproducibility as well as difference in signal to background. The “*z*” scores of >0.5 are indicative of the suitability of the assay for high-throughput screening and reflect good separation between the median plate values and that of the positive and negative controls (23). The optimization will require the screening of a small chemical library of at least 2,000 compounds in a 384-well format. The assay will need to be repeated at least seven times on different days, along with the determination of well-to-well and plate-to-plate variability.

## MATERIALS AND METHODS

### Reagents

A 20-nucleotide-long FRET RNA (5′FAM-CUCCUCAUUUUUCGCUAGUU-IB3′) labeled with 6FAM at the 5′ terminus and Iowa Black (IB) at the 3′ terminus was synthesized by IDT. MnCl_2_ and other reagents to prepare buffers were from Sigma. RNase A was from NEB (Cat# T3018L). RNasin was from Promega (Cat# N2111). Quick RNA mini prep kit was from Zymo (Cat # R1055). The 2,4-dioxo-4-phenylbutanoic acid (DPBA) was from Sigma (Cat # AMBH9884C268). GeneJET Plasmid Miniprep kit was from Thermo Scientific (Cat# K0503).

### Plasmid construction

The plasmid expressing the endonuclease domain of Sin Nombre virus (SNV) RdRp was constructed as previously reported (14, 20, 22). Briefly, the gene encoding the SNV RdRp was previously synthesized and cloned in pCDNA 3.1(+) backbone to generate the pCDPol construct (14, 20, 22). The DNA segment encoding the N-terminal endonuclease domain (250 amino acids) of the RdRp was PCR amplified from the pCDPol plasmid using two opposing primers. The PCR product was gel purified and cloned in the pTriEx1.1 backbone between NcoI and XhoI restriction sites to generate the plasmid ptPol1-250, which expresses C-terminally His-tagged endonuclease domain of SNV RdRp in *E. coli*. We used fusion PCR to generate the plasmid ptPol1-250D97A that expresses endonuclease mutant containing a point mutation (D97A) at the active site. Briefly, the region encoding the N-terminal 100 amino acids was PCR amplified using two opposing primers, and the mutation D97A was incorporated through the reverse primer. The region encoding the C-terminal 150 amino acids was also PCR amplified using two opposing primers. The two PCR products, having an overlap of approximately 20 nucleotides, were joined by fusion PCR, and the resulting PCR product was gel purified and cloned between NCO1 and XhoI restriction sites in pTriEx1.1 backbone, as previously reported (22). The same strategy was used in the cloning of SNV N protein in pTriEx1.1 backbone between NCOI and XhoI restriction sites to generate the ptSNVN construct that expresses C-terminally His-tagged SNV N protein in *E. coli*, as previously reported (14, 20, 22).

### Expression and purification of proteins

The wild-type SNV N protein, RdRp endonuclease domain, and its point mutant were expressed from ptSNVN, ptPol1-250, and ptPol1-250D97A plasmids, respectively, and purified as C-terminally His-tagged fusion proteins in bacteria as previously reported (14, 22). Purification was carried out by NiNTA chromatography on the AKTA pure protein purification system (GE Healthcare), using a native purification protocol as previously reported (22, 24). Briefly, *Escherichia coli* Rosetta (DE3) cells (Stratagene) were transformed with the plasmid of interest. The colonies expressing the protein of interest were grown in 500 mL of cultures at 37°C until the OD at 600 nm reached 0.5. The protein expression was induced by the addition of 0.5 mM isopropyl β-D-1-thiogalactopyranoside (IPTG) to the bacterial culture. The cultures were grown overnight at 18°C. Cells were harvested by centrifugation and re-suspended in lysis buffer (50 mM Tris-HCl, pH 7.4, 150 mM NaCl, 2 mM dithiothreitol [DTT], 0.5% Triton-X100, 5 mM CHAPS, 0.1 mM phenyl methyl sulfonyl fluoride [PMSF]). The re-suspended cells were briefly sonicated on ice and cleared by centrifugation. The cleared cell lysates were passed through 0.45 µM filters and loaded onto HisTrap NiNTA column having 5 mL of bed volume (Sigma), pre-equilibrated with the lysis buffer. The column was then washed with 50 mL of lysis buffer, followed by additional washing with 100 mL of wash buffer (50 mM Tris, 500 mM NaCl, 0.05% Triton X-100, and 20 mM Imidazole). The bound protein was finally eluted by imidazole gradient from 0 to 250 mM in the elution buffer (50 mM Tris, 500 mM NaCl, 0.05% Triton X-100), as previously reported (22, 24). The protein in eluted fractions was quantified and stored at −80°C.

### T7 transcription for the synthesis of 300-nucleotide-long test RNA

The DNA sequence encoding the 300-nucleotide-long test RNA was PCR amplified from pcDNA3.1(+) using a forward primer complementary to the plasmid sequence 20 nucleotides upstream of the T7 promoter and reverse primer complementary to the plasmid sequence 300 nucleotides downstream of the T7 promoter. The pcDNA3.1 (+) vector harbored the gene for CCHF virus S-segment mRNA. The resulting PCR product was gel purified and used as a template in an *in vitro* T7 transcription reaction. RNA synthesis was carried out using the T7 RiboMax kit (Promega), following the manufacturer’s instructions, as previously reported (17–19). The DNA template in the transcription reaction was digested using DNase I, followed by the purification of test RNA using the Quick RNA mini prep kit (Zymo) following the manufacturer’s instructions.

### Examination of RNA degradation by FRET assay

The degradation of FRET RNA by the purified endonuclease domain was examined by FRET Assay on a Shimadzu spectrofluorometer RF-5301PC. Briefly, the synthetic FRET RNA was dissolved in 350 μL of RNA digestion buffer (10 mM Tris HCl, pH 8.0, and 1 mM MnCl2) at required concentrations in the fluorescence glass cuvette (Starna cells). Fluorescence spectrum of FRET RNA from 495–650 nm was recorded in RNA digestion buffer using an excitation wavelength of 465 nm (excitation slit width, 5 nm, and emission slit width, 10 nm). The unaltered fluorescence signal of FRET RNA, in the absence of an endonuclease domain over time at 520 nm indicated the absence of photodegradation. The fluorescence spectra of FRET RNA (495–650 nm) were recorded after incubation with different concentrations of purified endonuclease domain, as mentioned in the text. All FRET studies were carried out at room temperature.
